# COVID-19 Vaccination and Acute Anterior Uveitis—A Case Control Study

**DOI:** 10.3390/vaccines13020176

**Published:** 2025-02-12

**Authors:** Asaf Shemer, Amit Toledano, Aya Altarescu, Biana Dubinsky-Pertzov, Assaf Rozenberg, Idan Hecht, Adi Einan-Lifshitz, Eran Pras

**Affiliations:** 1 Department of Ophthalmology, Shamir Medical Center (Formerly Assaf-Harofeh), Tzrifin 70300, Israel; amitoled@gmail.com (A.T.); ganonaya@gmail.com (A.A.); bianad@gmail.com (B.D.-P.); assafroz@gmail.com (A.R.); idanhe@gmail.com (I.H.); adi.einan@gmail.com (A.E.-L.); eranpras@gmail.com (E.P.); 2 Sackler Faculty of Medicine, Tel Aviv University, Tel Aviv 69978, Israel; 3 The Matlow’s Ophthalmo-Genetics Laboratory, Department of Ophthalmology, Shamir Medical Center (Formerly Assaf-Harofeh), Tzrifin 70300, Israel

**Keywords:** COVID-19, SARS-CoV-2, coronavirus, BNT162b2, vaccine, uveitis

## Abstract

Objective: To evaluate the association between the BNT162b2 (Pfizer-BioNTech^®^) coronavirus disease vaccine and new-onset anterior uveitis. Methods: A retrospective case control study of patients admitted and diagnosed with new-onset acute anterior uveitis, and matched controls admitted for other reasons (1:3 ratio), was completed. Rates of exposure to the BNT162b2 vaccine were compared between groups, and odds ratios for exposure to the vaccine were calculated. A secondary analysis of the overall number of patients with new-onset anterior uveitis in the six preceding years was conducted. This study was conducted in one academic center in Israel. Results: A total of 16 patients were admitted for acute anterior uveitis during the study period. Of the 16 cases, 11 (69%) received the first dose of the BNT162b2 vaccine prior to presentation and 8 (50%) also received the second dose. This compares to 39 (81.2%) in the control group. The odds ratio for exposure to the vaccine among cases was 0.508 (95% confidence interval 0.141–1.829, *p* = 0.300). Compared with preceding years, the rate of cases diagnosed with acute anterior uveitis in 2021 was similar to the six preceding years (mean 11.8 ± 3.4 cases). Conclusions: In this case control study and comparison with preceding years, we found no evidence to suggest an association between vaccination with the BNT162b2 (Pfizer-BioNTech^®^) COVID-19 vaccine and new-onset acute anterior uveitis.

## 1. Introduction

Severe acute respiratory syndrome coronavirus 2 (SARS-CoV-2) is a highly contagious virus that causes coronavirus-19 (COVID-19). Since its occurrence, millions of people have died of COVID-19 worldwide and many more have suffered from its complications [1]. COVID-19 infection has also been associated with the development of long-term autoimmune and inflammatory processes such as immune thrombocytopenic purpura, Guillain–Barre syndrome, Bells’ palsy, antiphospholipid syndrome, and more [2,3,4].

As of December 2020, several COVID-19 vaccines were approved with emergency authorization by the US Food and Drug Administration with high efficacy rates and good safety profiles [5]. Yet, the initial clinical trial included only about 40,000 participants. In light of the high morbidity and mortality, Israel and other countries worldwide initiated a massive vaccination campaign for the general population. Initially, Israeli adult citizens were vaccinated with the BNT162b2 (Pfizer-BioNTech) vaccine only. This m-RNA vaccine was given twice 21 days apart and the majority of Israel’s population (>4,000,000 m) was vaccinated. Due to the resurgence of COVID-19, the Israeli Ministry of Health campaigned for a third and fourth dose of the vaccination to the general adult population gradually [6].

Uveitis refers to a group of diseases characterized by intraocular inflammation. Uveitis affects all age groups and can lead to harmful visual impairment. As it is occasionally associated with systemic disorders, early diagnosis and treatment are warranted [7,8]. Vaccine-associated uveitis has been previously described in cases such as immunization for the hepatitis B virus (HBV), human papillomavirus (HPV), influenza virus, Bacille Calmette–Guerin (BCG), measles–mumps–rubella (MMR), and more. Although the mechanism is not fully elucidated, either the adjuvant’s secondary inflammation or antigenic mimicry are implicated [9].

Over the past few years, several studies have examined the immune response following BNT162b2 vaccination, including large cohort studies, with inconsistent results [10,11,12,13,14,15]. In this study, we aim to evaluate the association of new-onset anterior uveitis in patients recently vaccinated with the BNT162b2 (Pfizer-BioNTech) vaccine.

## 2. Methods

This study adhered to the tenets of the Declaration of Helsinki and was approved by the institutional review board (IRB) of the Shamir Medical Center. Given the retrospective nature of the study, a waiver of informed consent was granted by the IRB, as the research involved the use of anonymized patient data collected from medical records without direct patient involvement. This study was conducted in one academic referral center: Shamir Medical Center (formerly “Assaf Harofeh”). This is the fourth-largest governmental medical center in Israel. The center serves a diverse patient population, providing a robust setting for clinical research.

In this case control study, we investigated the association between exposure to the BNT162b2 vaccine (at least one dose) and acute anterior uveitis. All patients that were admitted to the emergency department (ED) of our academic medical center and were diagnosed with a first episode of anterior uveitis were classified as cases. Cases were collected from 1 January 2021 to 30 September 2021 via a hospital computerized system according to ICD-9 code (364.00—acute and subacute iridocyclitis, unspecified). We retrospectively reviewed each medical chart and manually recorded rates and timings of vaccination with the BNT162b2 vaccine. Patients older than 18 years were included, while patients with a previous uveitis episode were excluded. For controls, we blinded randomly matched patients admitted to our ED for any reason other than acute uveitis. Age, sex, and admission date within 48 h were matched in a 1:3 ratio between the groups. In both groups, the percentage of patients exposed to the BNT162b2 vaccine (first or second dose) at any time and within the previous 30 days was calculated and the odds ratio for exposure were compared with corresponding confidence intervals.

As a secondary analysis, all patients diagnosed with acute anterior uveitis during the same time period (January–September) in the six preceding years, before the introduction of the COVID-19 disease or vaccine, were extracted and the data were compared to the cases, which represents the number of patients with anterior uveitis in the same time period.

### 2.1. COVID-19 Vaccination Timeline

The US Food and Drug Administration (FDA) issued an emergency use authorization for the BNT162b2 (Pfizer-BioNTech^®^, Mainz, Germany) vaccine, received in December 2020. It was authorized for all individuals older than 16 years and was injected in two doses separated by a 21-day interval. Later, in August 2021, a full license was given for the BNT162b2 vaccine [13]. The Israeli national vaccination campaign began on 19 December 2020, and by 1 September 2021, approximately 85% of the adult population over the age of 20 were already vaccinated [6].

### 2.2. Statistical Analysis

Statistical analysis was performed using IBM SPSS Statistics 25 (IBM Corp. Armonk, NY, USA). Unless otherwise specified, data are presented as mean ± standard deviation (SD). Categorical variables were compared using the chi-square test. Clinical parameter distributions were tested for normality by the Shapiro–Wilk test. Independent T-tests were conducted for continuous variables with a normal distribution and the Mann–Whitney U test was used for variables with a non-normal distribution. The odds ratio (OR) for exposure to the vaccine was calculated with the corresponding 95% confidence interval. Absolute Risk Reduction (ARR) was calculated to quantify the change in risk of acute anterior uveitis associated with vaccination. ARR was determined by subtracting the risk in the study group from the risk in the control group. The Number Needed to Treat (NNT) was calculated as the reciprocal of the ARR. *p*-values less than 0.05 on a two-sided test were considered statistically significant.

## 3. Results

During the study period, a total of 16 patients were diagnosed with acute anterior uveitis in the ED. The mean patient age was 41.4 ± 19.6 years (range: 18–75 years) and 56.3% were female. The left eye was involved in six cases, the right in nine cases, and a bilateral disease was seen in one case.

On presentation, all patients had an anterior chamber reaction and, per our exclusion criteria, no vitreal, retinal, or choroidal involvement was seen. The disease was idiopathic in 14 cases, suspected to be associated with Behçet’s disease in 1 case, and was HLA-B27-associated in another. In one case, elevated intraocular pressure (IOP) was seen (31 mmHg), while, in the other cases, IOP was normal (<21 mmHg). Of the sixteen patients, three (18.7%) had diabetes mellitus, four (25%) had hypertension, one (6.2%) had a personal history of immune- or inflammatory-associated disorders (ankylosing spondylitis), and two (12.5%) had a family history of immune- or inflammatory-associated disorders (Behçet’s disease). None had a previous episode. A detailed description of the patients is provided in Table 1. Of the 16 cases, 11 (69%) received the first dose of the BNT162b2 vaccine prior to presentation (mean time after vaccine: 177 ± 85 days, range: 15–311 days) and 8 (50%) also received the second dose prior to presentation (mean time after vaccine: 166 ± 57 days, range: 85–230 days). Comparing fully vaccinated (50%, 8/16) to non-fully vaccinated patients (50%, 8/16) showed no statistically significant differences in age (48.0 ± 20.6 years vs. 34.8 ± 17.3, *p* = 0.190) or gender (50% males vs. 37.5%, *p* = 0.614).

For each patient admitted for an episode of acute anterior uveitis, three controls were randomly selected which were matched for age, sex, and date of admission. Among the controls, common reasons for admission were chest pain, abdominal pain, and syncope. Overall, 11 patients (68%) with acute anterior uveitis were previously vaccinated with the first dose of the BNT162b2 vaccine compared to 39 (81.2%) in the control group (Table 2). The unadjusted odds ratio (OR) for exposure to the vaccine among cases was 0.508 (95% confidence interval [95%CI] was 0.141–1.829, *p* = 0.300). When comparing fully vaccinated individuals (first and second dose), 8 patients (50%) with acute anterior uveitis were previously vaccinated compared to 75% (36/48) in the control group. In this case, the unadjusted OR for exposure to the vaccine among cases was 0.333 (95%CI was 0.103–1.082, *p* = 0.068). The Absolute Risk Reduction (ARR) for vaccination was calculated as 25% (ARR = 0.75–0.50). The Number Needed to Treat (NNT), defined as the number of individuals who need to be vaccinated to prevent a case of acute anterior uveitis, was 4 (NNT = 1/ARR).

Focusing on recently vaccinated patients (≤30 days), one case (6.2%, 1/16) with acute anterior uveitis was recently vaccinated with the first dose compared to four cases (8.3%) in the control group (OR: 0.733 95%CI: 0.076–7.08, *p* = 0.789). Furthermore, when comparing fully vaccinated individuals (first and second dose), no patient (0%) with acute anterior uveitis was recently vaccinated (≤30 days) compared to four patients (8.3%) in the control group (*p* = 0.233).

As a secondary analysis, a comparison of the overall number of patients with acute anterior uveitis was made to preceding years before the emergence of COVID-19 and the introduction of the BNT162b2 vaccine. Table 3 shows the number of cases in the six preceding years. Admission rates for acute anterior uveitis were similar in the preceding years compared to 2021 with a mean of 11.8 ± 3.4 cases (range: 8–17; Table 3).

## 4. Discussion

Uveitis may be associated with various systemic diseases. These diseases can be infectious and non-infectious. Of the non-infectious uveitis, systemic autoimmune or auto-inflammatory disease may be involved [12]. Among the autoimmune diseases, uveitis was found to be connected to sarcoidosis, HLA-B27 disease, Multiple Sclerosis, and more [14,15]. Multi Inflammatory Syndrome (MIS-C) associated with COVID-19 infection in children and young adults can present with a varied clinical spectrum. Ocular findings can include bilateral conjunctivitis (as part of Kawasaki-like disease). There are also reports of uveitis being the ocular-presenting symptom in COVID-19 patients [16,17].

COVID-19 vaccines have also been reported to be connected to autoimmune diseases, such as myocarditis, vasculitis, and Guillain–Barré syndrome, and even endothelial keratoplasty rejection. In addition, many ocular adverse effects have been reported so far, including cranial nerve palsies, acute macular neuroretinopathy, central serous chorioretinopathy, Vogt–Koyanagi–Harada reactivation, multiple evanescent white dot syndrome, Graves’ disease, and more. Yet, the vast majority of studies are retrospective case reports and case series, which cannot imply causality [18,19,20,21,22,23].

A retrospective population-based study, including over two million individuals who received the COVID-19 vaccine (with a matched cohort), reported a crude incidence rate of 47.1 per 100,000 person-years of non-infectious uveitis among vaccinated individuals compared to 50.6 per 100,000 in a historical non-vaccinated cohort. The adjusted hazard ratio was 0.91 (95% CI, 0.75–1.10, *p* = 0.33). The authors concluded that the study did not identify an increased risk of non-infectious uveitis following COVID-19 vaccination in individuals without a prior history of uveitis, providing reassurance regarding the vaccine’s overall safety [24]. Another retrospective population-based study that assessed the association between BNT162b2 vaccine and the risk of non-infectious uveitis was published. Overall, 188 cases were reported after the first or second dose of the vaccine. The authors reported an age- and sex-related standardized incidence ratio (SIR) of 1.41 with a 21-day attributable risk of 1.12 cases per 100,000 patients that were vaccinated and an SIR of 1.31 with an attributable risk of 0.85 per 100,000 patients following the first and second COVID-19 vaccine, respectively. The authors concluded that, although the BNT162b2 vaccine may be associated with non-infectious uveitis, their results do not provide proof for a cause–effect relationship [25].

In addition, one multicenter retrospective study was conducted to assess uveitis cases following the BNT162b2 mRNA vaccine. The authors found that cases of post-COVID-19-vaccine uveitis were mostly anterior and mild to moderate in severity with a mean time of 7.5 ± 7.3 days between vaccinations to uveitis onset. Two patients developed multiple evanescent white dot syndrome (MEWDS) and one patient had a complicated anterior uveitis, which eventually involved mild vitreal haze and macular edema. Approximately 40% of patients had experienced a previous uveitic episode, and in only two cases, both eyes were involved. All patients completed a two-dose vaccination with the BNT162b2 COVID-19 vaccine. Most patients achieved complete resolution (or significant improvement) at the final follow-up. The authors suggested a possible link between the vaccine and uveitis via type-I interferon secretion that induced autoimmune reactivity in prone subjects. Finally, the authors concluded that, although uncommon, there is a possible association between BNT162b2 vaccine and uveitis [26].

Furthermore, there are a few case reports exploring the connection between uveitis and the COVID-19 vaccine. In one case, a 23-year-old male developed anterior uveitis 14 days after the second dose of the BNT162b2 vaccine. Ocular inflammatory signs resolved following topical steroidal treatment [27]. In another case, a juvenile idiopathic arthritis (JIA)-associated anterior uveitis was documented in an 18-year-old female five days following the second dose of the BBIBP-CorV inactivated vaccine (Sinopharm^®^). The patient was treated topically with prednisolone acetate 1% with full resolution. The authors of these reports implied that this phenomenon may be a response to vaccine adjuvants [28]. Additional case reports of a 50-year-old woman diagnosed with bilateral uveitis five days following immunization with the inactivated COVID-19 vaccine and a 27-year-old male with juvenile idiopathic arthritis and positive HLA B-27 were described. The patients experienced a flare-up following the ChAdOx1 (COVISHIELD^®^) COVID-19 vaccination. In both cases, the patients fully recovered after topical steroidal treatment. The authors suggested an upregulation of the innate immune system as a potential mechanism [29,30]. However, given the mass vaccination in the population, it is likely that only some cases of uveitis will occur soon after vaccination completely by chance.

In this study, we looked at cases of anterior uveitis in naïve patients in one academic center. A matched case control design was used in addition to a secondary historical case series analysis. Our findings are in accordance with the result presented by Rabinovitch et al. and by Tomkins-Netzer et al. in terms of patient characteristics, laterality, and resolution following topical treatment [24,26].

Our study has limitations. First, our study includes a small sample size, which limits the statistical power of our findings. While the occurrence of uveitis in vaccinated individuals may seem significant, the relatively small cohort size means that our results should be interpreted with caution. Second, a passive surveillance system of vaccine-related adverse effects may not identify all cases. Furthermore, our study methodology included only patients who received the ICD-9 code upon discharge. Such a classification may underestimate the true number of the cases. Third, the data presented herein are from a large medical center. Some straightforward anterior uveitis cases may have been treated by the community care system and were not referred for hospital care. Fourth, we excluded all cases of recurrent uveitis and, therefore, we cannot comment regarding a potential flare-up following COVID-19 immunizations. Finally, in this initial report, we could only address patients’ initial episode and outcome; yet, we cannot assess long-term effects or disease course over time. It is important to emphasize that this study does not aim to establish a causal relationship between the COVID-19 vaccine and the occurrence of uveitis. Given the observational nature of the case control design, we can only suggest a potential association, and causality cannot be inferred from our findings. Further studies with larger sample sizes and more robust methodologies are needed to explore any possible causal link. In discussing the association between COVID-19 vaccination and uveitis, it is important to acknowledge that COVID-19 vaccines have been shown to be protective against acute COVID-19 symptoms [5] as well as some persistent symptoms [31]. Our study does not aim to question or diminish the protective benefits of vaccination, which remain well established. We emphasize that our findings should not be misinterpreted as supporting any anti-vaccine agenda.

In conclusion, this study could not identify an association between the BNT162b2 vaccine and a new acute onset of anterior uveitis. Further multicenter prospective investigations are warranted to create a better understanding of the BNT162b2 vaccine effects. In our opinion, vaccine-related adverse events should be monitored and reported in order to allow the patient and the physician a better understanding of a newly authorized vaccine. However, in times of an acute global lethal pandemic, effective immunization is currently the most important action the public should carry out.

## Figures and Tables

**Table 1:** Characteristics of patients with new-onset anterior uveitis after recent vaccination with the BNT162b2 (Pfizer-BioNTech) vaccine.

Patient	Gender	Age	Comorbidities	Laterality	AC Cells *	AC Flare *	IOP *	Etiology	Follow-Ups	Status at Last Follow-Up	Topical Steroids (Drops per Day)	Topical Mydriasis	Topical Cycloplegia	Recovery Time (Days)
1	M	42	None	Both	1	0	12	Idiopathic	5	Partially recovered	4	3	0	21
2	F	52	Cataract	R	1.5	0	16	Idiopathic	3	Fully recovered	6	0	2	44
3	M	73	Cataract, DMT2, HTN, IHD	R	2	0	12	Idiopathic	1	Partially recovered	6	0	0	4
4	M	39	S/P perimyocarditis	L	3	1	10	Idiopathic	0	N/A	12	0	3	N/A
5	F	48	Ankylosing spondilitis, HTN, S/P bariatric surgery	R	2.5	2.5	8	Ankylosing spondilitis	3	Fully recovered	6	0	3	46
6	F	27	Atopic dermatitis	R	2	0	10	Idiopathic	1	Partially recovered	8	3	0	3
7	M	28	Family history of Behçet’s disease	R	2	1	12	m/p Behçet’s	3	Fully recovered	8	0	3	120
8	F	26	None	L	4	2	8	Idiopathic	3	Fully recovered	12	0	2	28
9	F	67	Cataract, DMT2, HTN, Parkinson’s disease	L	1	0	10	Idiopathic	4	Fully recovered	12	0	3	85
10	F	18	Axenfeld-Riger syndrome, psoriasis, f/h Behçet’s disease	L	3	0	31	Behçet’s	2	Fully recovered	6	0	0	68
11	F	18	None	L	1.5	0	9	Idiopathic	3	Fully recovered	12	0	0	22
12	M	19	None	R	1	0	12	Idiopathic	0	N/A	0	0	0	N/A
13	F	24	None	R	2	1	16	Idiopathic	1	Fully recovered	6	0	2	4
14	F	53	None	L	2	1	8	Idiopathic	2	Fully recovered	12	0	2	24
15	M	54	None	R	1	0	12	Idiopathic	1	N/A	4	0	3	3
16	M	75	Cataract, HTN, dyslipidemia	R	2	0	14	Idiopathic	0	N/A	4	0	0	N/A

* At initial presentation. Abbreviations: AC, anterior chamber; DMT2, diabetes mellitus type 2; F, female; F/h, family history; HTN, hypertension; IHD, ischemic heart disease; L, left; M, male; M/p, most probably; N/A, not available; R, right; S/P, status post.

**Table 2:** Distribution of vaccinated and non-vaccinated patients among cases with new-onset anterior uveitis and matched controls.

Patient Group	No. of Cases	No. of Controls	Total No.
Vaccinated	11	39	50
Non-vaccinated	5	9	14
Total	16	48	64

**Table 3:** Cases with acute anterior uveitis admitted in 2021 and during the same period in the six preceding years.

Year	Cases	Age (Years)	Male Gender
2015	15	46.2 ± 16.5	8 (53%)
2016	8	45.1 ± 17.9	5 (62%)
2017	11	47.9 ± 20.1	4 (36%)
2018	17	45.7 ± 16.7	10 (59%)
2019	10	51.9 ± 15.9	5 (50%)
2020	10	39.4 ± 20.0	7 (70%)
2021	16	41.4 ± 19.6	7 (44%)

## Data Availability

The data supporting the findings of this study are not publicly available due to privacy and ethical considerations.

## Correction Statement

Following the removal of this article from the Special issue COVID Vaccines: Design, Development, and Immune Response Studies: 2nd Edition, an Editorial Board member reviewed the entire editorial process and confirmed all elements had been carried out as per MDPI Editorial Process policy (https://www.mdpi.com/editorial_process). As a result, the original Academic Editor listed on this publication has been replaced with that of the Editorial Board member who conducted this review. The original publication has now been updated accordingly. This information has also been transmitted to relevant indexing organizations.
